# Genetic Characterization of *Trypanosoma cruzi* DTUs in Wild *Triatoma infestans* from Bolivia: Predominance of TcI

**DOI:** 10.1371/journal.pntd.0001650

**Published:** 2012-05-29

**Authors:** Simone Frédérique Brenière, Claudia Aliaga, Etienne Waleckx, Rosio Buitrago, Renata Salas, Christian Barnabé, Michel Tibayrenc, François Noireau

**Affiliations:** 1 MIVEGEC (Université de Montpellier 1 et 2, CNRS 5290, IRD 224), Maladies Infectieuses et Vecteurs: Ecologie, Génétique, Evolution et Contrôle, Institut de Recherche pour le développement (IRD), Representation in Bolivia, La Paz, Bolivia; 2 Instituto Nacional de Laboratorios de Salud (INLASA), Laboratorio de Entomologia Médica, La Paz, Bolivia; 3 IIBISMED, Facultad de Medicina, Universidad Mayor de San Simón, Cochabamba, Bolivia; Universidad de Buenos Aires, Argentina

## Abstract

**Background:**

The current persistence of *Triatoma infestans* (one of the main vectors of Chagas disease) in some domestic areas could be related to re-colonization by wild populations which are increasingly reported. However, the infection rate and the genetic characterization of the *Trypanosoma cru*zi strains infecting these populations are very limited.

**Methodology/Principal Findings:**

Of 333 wild *Triatoma infestans* specimens collected from north to south of a Chagas disease endemic area in Bolivia, we characterized 234 stocks of *Trypanosoma cruzi* using mini-exon multiplex PCR (MMPCR) and sequencing the glucose phosphate isomerase (*Gpi*) gene. Of the six genetic lineages (“discrete typing units”; DTU) (TcI-VI) presently recognized in *T. cruzi*, TcI (99.1%) was overdominant on TcIII (0.9%) in wild Andean *T. infestans*, which presented a 71.7% infection rate as evaluated by microscopy. In the lowlands (Bolivian Chaco), 17 “dark morph” *T. infestans* were analyzed. None of them were positive for parasites after microscopic examination, although one TcI stock and one TcII stock were identified using MMPCR and sequencing.

**Conclusions/Significance:**

By exploring large-scale DTUs that infect the wild populations of *T. infestans*, this study opens the discussion on the origin of TcI and TcV DTUs that are predominant in domestic Bolivian cycles.

## Author Summary

Chagas disease is a neglected parasitic disease transmitted by bugs (vectors) and represents a serious health problem in the Americas. Although the transmission generally occurs in the houses where the bugs are living, wild populations of vectors are now considered a problem because these populations might enter the houses and recolonize them after eliminating of house populations by insecticide spraying. This is the case of the Southern countries where *Triatoma infestans*, the principal vector, transmits *Trypanosoma cruzi* the agent of the disease. This parasite presents a large genetic variability and it is important to know which *T. cruzi* genotypes are carried by the vectors. The authors found that in the wild *T. infestans* from the Bolivian Andean region, a principal group of genotype was circulating. In the lowlands (Bolivian Chaco), another additional genotype group was detected. Together with exploring at large scale which genotypes are infecting *T. infestans* wild populations, this study opens the discussion on the origin *T. cruzi* genotype groups. Also this study completes our basic knowledge on *T. cruzi* subspecific genetic variability, and therefore brings new tools for molecular epidemiology of Chagas disease.

## Introduction

*Trypanosoma cruzi*, the agent of Chagas disease, is a serious threat to health in the Americas, accounting for the highest disease burden in Latin American, with eight to nine million people infected and 25–90 million at risk [1]–[3]. This parasite, which belongs to the order Kinetoplastida, is mainly transmitted by blood-sucking bug vectors (Hemiptera, Reduviidae, Triatominae) but also by blood transfusion and oral transmission. Moreover, newborns can be infected through vertical transmission. There are currently 141 recognized species of triatomines, but only five of them, belonging to three genera (*Triatoma*, *Rhodnius*, and *Panstrongylus*) can be considered important vectors of Chagas disease [4]. With the exception of one species (*T. rubrofasciata*), all Triatominae have populations living in natural habitats in contact with wild mammals, birds, or reptiles [5]–[8]. *T. cruzi* is found in three overlapping ecosystems. One is related to the wild environment and involves wild populations of triatomines and mammals (sylvatic cycle); the second one depends on artificial structures surrounding human dwellings where vector populations associated to domestic and synanthropic animals live (peridomestic cycle); the third one occurs in dwellings and involves triatomines living indoors, humans, and domestic animals (domestic cycle).

Population genetics analyses have shown that *T. cruzi* has a predominantly clonal mode of evolution and exhibits considerable phenotypic and genetic diversity [9]. This population genetics model refers to genetic clonality, i.e., limited or absent genetic recombination with persistence of durable multilocus associations, whatever the cytological mechanism of reproduction [10]. Six distinct genetic lineages or discrete typing units (DTUs) [11] have been described [12], [13]. They have recently been validated by a committee of experts and labeled TcI to TcVI [14]. TcI is ubiquitous and prevalent in different sylvatic cycles. However, it is responsible for the large majority of human infections in the Amazon basin and more northern countries as well as part of the infections in South Cone countries of South America. It exhibits considerable genetic diversity [9], [15], [16] with possible subclustering [17], [18]. TcII, V, and VI are mainly associated with domestic cycles and prevalent in human infections in the Southern Cone countries; TcV and TcVI are hybrid genotypes, whose putative ancestors are TcII and TcIII [19], [20]. Finally, TcIII and IV are more rarely sampled throughout the endemic area and seem to be specific to sylvatic cycles, with few reports of human infection.

In Bolivia, *Triatoma infestans* (Hemiptera: Reduviidae) remains the main domestic vector of *T. cruzi*. It is the target of the National Control Program based on house-spraying with residual insecticides. Wild populations of *T. infestans* are now seriously considered a problem to keep the villages free of triatomines [21]–[23]. Sylvatic populations of the vector have been described in different Andean valleys in Bolivia [21], [22], [24], [25]. Moreover, the detection of wild foci of *T. infestans* in the Bolivian Chaco has extended the distribution of wild populations to the lowlands of Bolivia [26].

Two main genotypes belonging to TcI and TcV were previously identified in the domestic cycle in regions where *T. infestans* was the vector [27]–[33]. Moreover, these genotypes had been identified in strict sympatry in the same host [27], [34]. In contrast, few data are available on DTUs circulating in sylvatic *T. infestans*. Dujardin et al. [35] found that wild *T. infestans* were infected with the same *T. cruzi* genotypes as domestic *T. infestans* (TcI and TcV), with the same frequencies. They took this as additional evidence of a lack of speciation between wild and domestic *T. infestans*. Another study identified TcI as the only DTU in a wild focus located in the valley of Cochabamba, Bolivia [25].

Among the genetic markers that can identify the different *T. cruzi* groups the non-transcribed spacer region of the mini-exon gene was previously proposed to discriminate *T. cruzi* I (now TcI), *T. cruzi* II (now TcII), *T. cruzi* zymodeme 3 (now TcIV), using the mini-exon multiplex PCR (MMPCR) [36]. Recently, the MMPCR analysis was applied on a large sample of stocks (previously characterized by multilocus typing) belonging to the six DTUs, showed 3 DTU tags: a 200 bp PCR product for TcI, a 250 bp for TcII, TcV and TcVI and a 150 bp for TcIII and TcIV [37]. This method was also successfully applied for rapid DTU identification in triatomine digestive tracts [5], [38]. Moreover, among housekeeping genes, the glucose-6-phosphate isomerase (*Gpi*), a single-copy nuclear gene, presented a sequence polymorphism that is valuable for characterization of DTUs [19], [39].

In this study, we applied the MMPCR and *Gpi* sequencing for the characterization of *T. cruzi* DTUs directly in the digestive tract of wild *T. infestans* collected in Bolivia.

## Materials and Methods

### Origin of *T. infestans* populations

**Figure 1:**
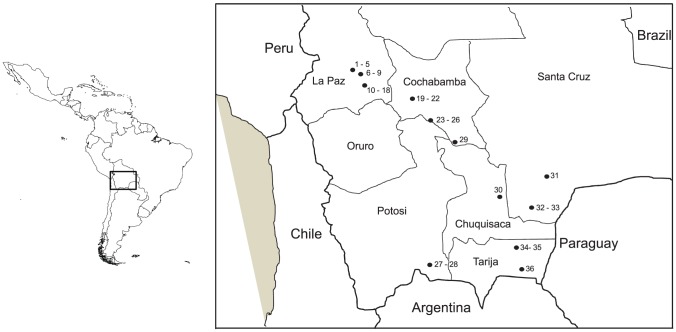
Sampling sites of wild populations of *Triatoma infestans* in Bolivia. The sites were numbered from 1 to 36, Bolivian department names are indicated, for the DTU *T. cruzi* results see in Table 1.

The triatomines were sampled in sylvatic environments from April to November 2009 (Figure 1). Collections were carried out using mice-baited adhesive traps [40] in different ecotopes such as under bush and bromeliads, rocks, burrows, hollow trees, and stone walls. The bugs were transported alive to the laboratory for species confirmation using morphological taxonomic keys [41]. Table 1 summarizes the geographical and ecotope origin of the collected *T. infestans* according to the ecoregions defined in [42]. Briefly, the majority of the bugs were collected in Andean valleys where sylvatic foci have been previously described [21], [24] and the others were collected from new foci in the Bolivian Chaco where the “dark morph” type of *T. infestans* was discovered [22], [26]. Before dissection, feces from each bug were examined for the presence of trypanosomatid by direct microscopic observation at ×400 magnification (mo). Bugs were then dissected under a safety hood, and the digestive tracts stored at −20°C.

**Table 1:** Geographical origin, ecotopes of wild triatomines and *T. cruzi* identification by MMPCR.

Geographical origin								DTU groups of *T. cruzi* d		
N° site	Ecoregion^a^	Latitude (S)	Longitude (W)	Alt. (m)	Area	Ecotopes^b^	No. of specimens^c^	TcI	TcII, TcV, TcVI	TcIII, TcIV
1	BSIA	16°41′21.0″	68°00′40.9″	2679	A	1	5	5		
2	BSIA	16°42′08.0″	68°00′16.3″	2821	A	2	2	1		
3	BSIA	16°42′42.9″	67°59′25.3″	2732	A	3, 4	23	21		
4	BSIA	16°42′56.3″	67°52′13.6″	2459	A	3, 5	6	5		
5	BSIA	16°43′11.1″	67°52′26.5″	2380	A	3, 6, 7	11	9		
6	BSIA	16°48′51.5″	67°42′18.3″	1873	A	1, 6, 7, 8	19	19		
7	BSIA	16°49′43.3″	67°42′17.0″	1957	A	1, 7, 9	19	19		
8	BSIA	16°53′12.2″	67°42′43.1″	2095	A	7	13	13		
9	BSIA	16°55′48.8″	67°41′32.9″	2182	A	4, 8	18	16		
10	BSIA	17°00′30.1″	67°39′25.2″	2356	A	4, 6, 7	47	43		
11	BSIA	17°01′54.8″	67°40′38.6″	2159	A	2	2	1		
12	BSIA	17°03′55.6″	67°39′51.4″	2619	A	1, 3, 5, 7	5	5		
13	BSIA	17°04′07.7″	67°39′25.5″	2645	A	4, 7	5	5		
14	BSIA	17°04′24.2″	67°38′42.7″	2543	A	7	1	1		
15	BSIA	17°04′25.2″	67°37′59.7″	2493	A	6, 7, 10	5	5		
16	BSIA	17°04′44.6″	67°37′57.0″	2602	A	6, 7, 9	17	16		
17	BSIA	17°07′32.0″	67°35′59.5″	2864	A	7, 11	6	3		
18	BSIA	17°08′10.8″	67°35′17.9″	2767	A	3, 4, 6, 9	6	4		
19	BSIA	17°42′45.2″	66°29′38.8″	2583	A	7	9	9		
20	BSIA	17°25′39.2″	66°15′32.1″	2576	A	4, 7	11	11		
21	BSIA	17°27′45.5″	66°18′51.0″	2543	A	7	3	3		
22	BSIA	17°28′37.5″	66°08′16.1″	2710	A	7	14	4		
23	BSIA	17°59′16.4″	65°50′11.1″	2059	A	7, 8	10			1
24	BSIA	18°00′44.5″	65°48′32.6″	2025	A	7	4	3		
25	BSIA	18°01′50.9″	65°47′18.7″	1968	A	8	11			1
26	BSIA	18°06′21.0″	65°45′31.3″	2571	A	8	5			
27	PP	21°37′16.8″	65°48′46.0″	2963	A	6	7	7		
28	PP	21°44′51″	65°49′26.0″	3080	A	4	30			
29	CS	18°35′52.6″	65°07′33.4″	1754	I^g^	3, 7	1			1
30	BTB	19°55′39.2″	63°54′08.8″	1039	I^g^	uk^e^	1	1		
31	GC	19°21′20.8″	62°34′10.2″	398	N-A	12, uk^f^	3			
32	GC	20°11′10.1″	63°61′21.7″	614	N-A	12	2		1^h^	
33	GC	20°15′09.1″	62°58′26.9″	573	N-A	12	6			
34	GC	21°09′25.3″	63°22′30.7″	488	N-A	12	1			
35	GC	21°22′37.0″	63°21′34.8″	351	N-A	12	2	1^h^		
36	GC	21°50′50.6″	63°14′51.6″	443	N-A	12	3			
						Total	333	229	1	3

a The ecoregions are according to Ibisch et al. (2008), BSIA Bosque Seco Inter Andino, PP Prepuna, CS Chaco Serrano, BTB Bosque Tucumano Boliviano, GC Gran Chaco;

b 1 crack, 2 cliff, 3 under vegetation, 4 burrow, 5 adobe wall, 6 stone wall, 7 under stones, 8 cave, 9 hollow ground, 10 bird nest, 11 graves, 12 hollow tree, uk unknown;

c Total number of specimens tested by MMPCR;

d DTUs Discrete Typing Unit were identified by MMPCR, the undetermined samples were MMPCR negative;

e Specimen captured on the exterior wall of a house;

f Captured by light traps.

g Between Andean and non-Andean areas.

h These specimens were dark morphs.

### Mini-exon multiplex PCR (MMPCR)

DNA was extracted from triatomine digestive tracts with the QIAamp DNA mini kit (Quiagen, Courtaboeuf, France), according to the blood sample protocol. The multiplex primer set was as previously described: three oligonucleotides derived from the hypervariable region of *T. cruzi* mini-exon repeats, and a common downstream oligonucleotide, corresponding to sequences present in the best conserved region of the mini-exon gene used as opposing primer in the multiplex reaction. PCR conditions were according to Fernandes et al. [36], with slight modifications. DNA was amplified in a 25 µl reaction volume containing 1× reaction buffer, 1.5 mM MgCl^2^, 50 µM of each nucleotide, 0.2 µM of each primer, 0.5 UI of Taq polymerase (Roche Applied Science, Penzberg, Germany). The amplifications were performed in a thermocycler (Eppendorf, Hamburg, Germany), in previously described conditions [36]. PCR products were separated on 3% agarose gel using the molecular weight marker Smart Ladder (Eurogentec, Angers, France) and visualized under UV with Ez-vision (Amresco, Solon, OH, USA). The discrimination between DTUs was according to Aliaga et al. [37].

### PCR of the *T. cruzi* glucose-6-phosphate isomerase fragment

A 652 bp fragment was amplified with a set of primers, forward (*Gpi*-L) starting at position 591 of the gene (5′-CGCCATGTTGTGAATATTGG-3′) and reverse (*Gpi*-R) starting at position 1246 (5′–TTCCATTGCTTTCCATGTCA-3′), from a subsample of 15 DNAs which had given an intense MMPCR band. DNA was amplified in a 25 µl reaction volume containing 0.75 mM MgCl^2^, 0.2 mM of each nucleotide, 0.4 µM of each primer, 2.5 UI of Taq DNA polymerase (Roche Applied Science, Penzberg, Germany), and 20 ng of DNA template. The amplification took place in a thermocycler (Eppendorf, Hamburg, Germany), with the following cycle conditions: 94°C for 3 min; 94°C for 1 min, 58°C for 1 min, and 72°C for 1 min (35 cycles); 72°C for 5 min. Purification and direct sequencing of both strands of DNA amplicons were performed by the company MACROGEN (Seoul, South Korea). Sequences were aligned and corrected using BioEdit software v. 7.0.9 [43], and a 458 bp partial sequence was resolved for each sequence (from nucleotide site 691–1148).

## Results

### Mini-exon multiplex PCR (MMPCR) analysis

**Figure 2:**
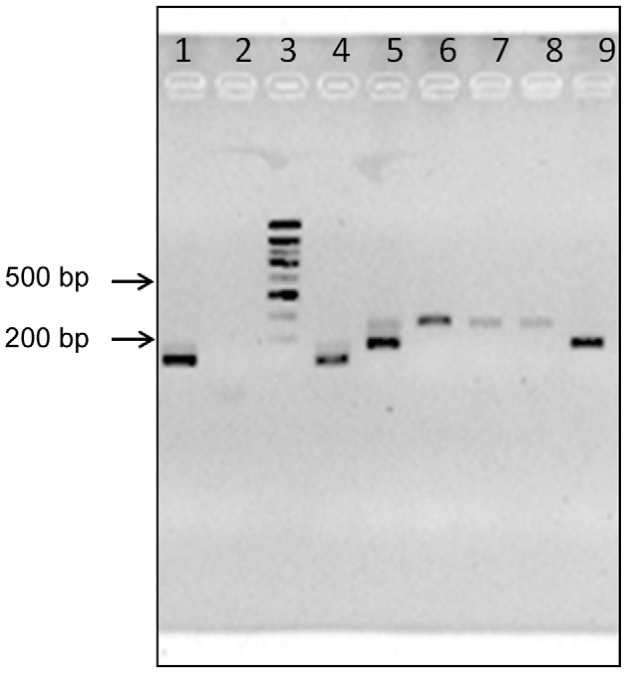
Illustrating electrophoresis of MMPCR products. Ez-vision stained 3% agarose gel containing MMPCR products obtained from DNA extracts of reference strains and current digestive tracts of *T. infestans*. Lane 1, sample Tor05; lane 2, PCR negative sample; lane 3, the molecular weight marker Smart Ladder (Eurogentec, Angers, France); lane 4–6, reference strains (M6241cl6, P209cl1 and MNcl2 respectively); lane 7–8, sample Char09 (two independent PCR); lane 9, sample Lur112. See Table 2 for DTUs information.

A total of 333 DNA samples from digestive tracts of wild *T. infestans* were processed in MMPCR for DTU identification. Among them 20.1% were adults of both sexes, 64.1% 4^th^ and 5^th^ instar nymphs, and 15.8% 2^nd^ and 3^rd^ instar nymphs. Before dissection, the bug feces were examined (85.0% of the total sample) using microscopy. The parasite infection rate was 71.7% in Andean specimens while no positive insect was found among the 17 specimens from Chaco (GC ecoregion). The accordance between microscopic observation and MMPCR was 82%, with 93.1% of positive MMPCR when mo was positive and 17,5% when mo was negative. The identification of the three DTUs was assessed by determining the molecular weight of the MMPCR products for each sample (Table 1, Figure 2). The results showed that the large majority (98.3%) of the 234 wild *T. infestans* specimens were infected by TcI (PCR products of 200 bp). Only one sample from an adult *T. infestans* (“dark morph” type) collected at site 32 (GC ecoregion) gave a 250 bp MMPCR product corresponding to TcII, TcV, or TcVI. Three other samples (sites 23, 25, and 29) gave a 150 bp MMPCR products corresponding to either TcIII or TcIV. The MMPCR product of the specimen of the latter group captured at site 29 was sequenced and the DNA fragment (64 bp) matched the TcIII reference stock named M5631 (accession No AF050521.1 and AY367126.1, 98% identity).

### Sequence variability of the *Gpi* gene from *T. cruzi* infecting wild *T. infestans*

A partial sequence of the *Gpi* obtained from 15 samples (13 from the set corresponding to TcI, one from the set corresponding to TcII, TcV, or TcVI , and one from the set corresponding to either TcIII or TcIV) were sequenced in order to explore the variability within TcI and to discriminate the DTUs within the other sets. The 458 bp partial sequences (starting at site 691 and ending at site 1148 of the entire CL Brener stock gene, accession no. XM815802.1) were aligned with the sequences corresponding to *T. cruzi* reference stocks belonging to the six DTUs previously deposited in GenBank (Table 2). With no ambiguity, each sequence under study had been attributed to a DTU. Within TcI, 3 sequences were observed: the most frequent (11 stocks) presented 100% identity with the two identical sequences from TcI reference stocks (OPS21 and P/209) deposited in GenBank; the two other sequences exhibited a single mutation and the Vis01 stock identified in a triatomine bug captured at site 27, presented a heterozygous pattern at nucleotide position 940. The sequence of the Char09 of the second set (corresponding to TcII, TcV, or TcVI), detected in a “dark morph” (site 32), presented 100% identity with two identical TcII reference stocks (Tu18cl2 and CBBcl3). For the sample of the last set corresponding to either TcIII or TcIV (Tor05 from site 25), the sequence presented 100% identity with two identical TcIII reference stocks (M6241cl6 and X110/8).

**Table 2:** Variable sites of glucose phosphate isomerase gene of *T. cruzi* identified in wild *T. infestans* compared with reference stocks.

Name	Accession no.	DTU^a^	No. of current stock	Country	Area^b^	Nucleotide position																			
						715	739	784	805	811	828	829	830	831	832	856	859	863	898	913	940	945	1030	1051	1108
OPS21	AY484472	TcI		Venezuela		A	A	A	T	T	G	T	G	A	G	G	C	C	A	G	T	C	G	C	T
P/209cl1	AY484473	TcI		Bolivia		.	.	.	.	.	.	.	.	.	.	.	.	.	.	.	.	.	.	.	.
Aiq02^C^	JN653335	TcI	1	Bolivia	A	.	.	.	.	.	.	.	.	.	.	.	.	.	.	.	C	.	.	.	.
Lur 112^c^^,^ d	JN653324-34	TcI	11	Bolivia	A	.	.	.	.	.	.	.	.	.	.	.	.	.	.	.	.	.	.	.	.
Vis01c	JN653336	TcI	1	Bolivia	A	.	.	.	.	.	.	.	.	.	.	.	.	.	.	.	Y	.	.	.	.
Tu18cl2	AY484477	TcII		Brazil		.	T	.	C	C	A	.	.	.	.	.	T	T	G	.	.	A	.	G	.
CBBcl3	AY484476	TcII		Chile		.	T	.	C	C	A	.	.	.	.	.	T	T	G	.	.	A	.	G	.
Char09^c^	JN653338	TcII	1	Bolivia	NA	.	T	.	C	C	A	.	.	.	.	.	T	T	G	.	.	A	.	G	.
M6241cl6	AY484478	TcIII		Brazil		.	.	.	.	.	.	.	.	.	.	A	.	.	.	.	.	.	.	.	.
X110/8	AY484479	TcIII		Paraguay		.	.	.	.	.	.	.	.	.	.	A	.	.	.	.	.	.	.	.	.
Tor05^c^	JN653337	TcIII	1	Bolivia	A	.	.	.	.	.	.	.	.	.	.	A	.	.	.	.	.	.	.	.	.
CanIIIcl11	AY484474	TcIV		Brazil		.	.	C	.	.	.	.	.	.	.	.	.	.	G	A	.	.	T	T	C
EP272	AY484475	TcIV		Colombia		.	.	C	.	.	.	.	.	.	.	.	.	.	G	A	.	.	T	T	.
MNcl2	AY484480	TcV		Chile		.	W	.	Y	Y	R	K	R	R	K	.	Y	.	G	.	.	M	.	S	.
Bug2148cl11	AY484481	TcV		Brazil		.	W	.	Y	Y	R		R	R		.	Y	.	G	.	.	M	.	S	.
ClBrener	AY484482	TcVI		Brazil		R	W	.	Y	Y		K	R	R	K	.	.	.	R	.	.	.	.	.	.
TulaCl2	AY484483	TcVI		Chile		R	W	.	Y	Y	R	K	R	R	K	.	.	.	G	.	.	M	.	S	.

a DTU, Discrete Typing Unit;

b A for Andean, NA for Non-Andean (lowland);

c Samples under study;

d Ten other samples had identical sequence, they were from Northern Andean area.

## Discussion

Recently, an active search for new foci of wild *T. infestans* in Bolivia enabled us to show that their distribution was broader than initially described [21], [44]. Also, few data on the genetic characterization of *T. cruzi* stocks infecting these vector populations were available, apart from the work by Dujardin et al ([35], conducted using multilocus enzyme electrophoresis, and the detection of the only TcI at Cotapachi 15 km west of Cochabamba city (Andean area) [25]. In the present context, where wild *T. infestans* highly infected can enter houses and recolonize them after domestic populations have been eliminated by insecticide spraying, it is important to know which *T. cruzi* DTUs are carried by the vectors. In this study, 234 *T. cruzi* stocks isolated from wild *T. infestans* were characterized by MMPCR. The vectors came from several areas mainly situated in two ecoregions in Bolivia, the Inter-Andean Dry Forest and the Gran Chaco where the “dark morph” was found. Regarding the detection of parasites in bugs, the correlation between detection of infection by microscopy (mo) and by the method of MMPCR was high (82%). However, some infected bugs (mo positive) were MMPCR negative probably due to the presence of inhibitors factors of the polymerase in the DNA extracts. At the contrary, several samples mo negatives were MMPCR positive, which allowed us to detect and identify few strains in dark morph specimens. In the overall sample, the TcI DTU is widely dominant, but in the Andean and intermediate areas TcIII stocks were detected. In the lowlands, only TcI and TcII were characterized in the “dark morph” specimens.

Interestingly, the DTU distribution in wild *T. infestans* is very different from that reported in domestic *T. infestans* collected before the vector control campaigns undertaken on a large scale in Bolivia since 2003; the frequencies of TcI only, TcV only, and mixed infections (TcI and TcV) were 38.6%, 16.8% and 32.7% respectively [31]. At the same time, TcV was mostly detected in patients during the chronic phase of the infection while both TcI and TcV were detected in younger patients with early infection [28], [45]. As for the vectors, it was suggested that the domiciliation of *T. infestans* had taken place in high Andean valleys and that the dispersal of domestic *T. infestans* to other areas had occurred by human transport [46], [47]. The current observations do not fit these hypotheses, since the only TcI (and to a lesser extent TcIII) would then have been introduced into domestic cycles but not TcV, unless it is assumed that TcV disappeared from the wild *T. infestans* cycle in the Andes valleys after its domiciliation.

Among the six *T. cruzi* DTUs , TcV and TcVI are composed of stocks that appear to be recent hybrids between TcII and TcIII [19]. Consequently, it is tempting to speculate that they might have arisen in an area where the putative parental DTUs coexist. Moreover, this hybridization event is still considered to have occurred much earlier than human colonization in South America [48]. Consequently, parental and hybrid DTUs are likely to coexist in the sylvatic cycle in a putative geographical area in South America. Lately, the Andean origin of *T. infestans* was challenged by the hypothesis of Chaquean origin [26], [44], [49], [50]. If parental and hybrid DTUs are not found in the sylvatic cycle in the Andes, an alternative might be the Gran Chaco region. These is no information regarding the genetic characterization of *T. cruzi* in the sylvatic cycle at the Bolivian lowlands, except for a report of a TcVI stock isolated from a *Didelphis marsupialis* specimen captured on the Amazon slope [51]. In the Paraguayan Chaco, TcII, TcIII and TcV have been identified in different wild mammal species [52] and in the Argentinean Chaco TcI was identified in *Didelphis albiventris* and TcVI in one *Conepatus chinga* [53]. In spite of fairly scarce data, the hypothesis that hybrid DTUs may have originated in Chaco should be considered, especially considering the detection of all DTUs except for TcIV in the domestic cycle in the Bolivian Gran Chaco (unpublished data). The search for DTUs circulating in sylvatic cycles will provide more valid information on the evolution of *T. cruzi* than studies conducted in domestic cycles where the geographical distribution of the DTUs is skewed by passive transport of parasites (human migration, triatomine transports) and by the selection of specific DTUs by hosts, considering that host diversity is lower in the domestic cycle than in sylvatic cycles.
